# The FDA “Black Box” Warning on Antidepressant Suicide Risk in Young Adults: More Harm Than Benefits?

**DOI:** 10.3389/fpsyt.2019.00294

**Published:** 2019-05-03

**Authors:** Michele Fornaro, Annalisa Anastasia, Alessandro Valchera, Alessandro Carano, Laura Orsolini, Federica Vellante, Gabriella Rapini, Luigi Olivieri, Serena Di Natale, Giampaolo Perna, Giovanni Martinotti, Massimo Di Giannantonio, Domenico De Berardis

**Affiliations:** ^1^Neuroscience, Reproductive Science and Odontostolmatology, Section of Psychiatry, University School of Medicine Federico II, Naples, Italy; ^2^Polyedra Research Group, Teramo, Italy; ^3^Alma Mater S.P.A. Villa Camaldoli, Naples, Italy; ^4^Villa S. Giuseppe Hospital, Hermanas Hospitalarias, Ascoli Piceno, Italy; ^5^NHS, Department of Mental Health, Psychiatric Service of Diagnosis and Treatment, Hospital G. Mazzini, ASL Teramo, Teramo, Italy; ^6^School of Life and Medical Sciences, University of Hertfordshire, Hatfield, Herts, United Kingdom; ^7^Hermanas Hospitalarias, FoRiPsi, Department of Clinical Neurosciences, Villa San Benedetto Menni, Como, Italy; ^8^Department of Neurosciences and Imaging, Chair of Psychiatry, University G. D’Annunzio, Chieti, Italy

**Keywords:** FDA, antidepressant, suicide, “black box” warning, Major depression (MDD), Bipolar Disorder

## Abstract

The decision made in the year 2004 by the U.S. Food and Drug Administration (FDA) to require a boxed warning on antidepressants regarding the risk of suicidality in young adults still represents a matter of controversy. The FDA warning was grounded on industry-sponsored trials carried one decade ago or earlier. However, within the past decade, an increasing number of reports have questioned the actual validity of the FDA warning, especially considering a decline in the prescription of the antidepressant drugs associated with an increase in the rate of suicidal events among people with severe depression. The present report provides an overview of the FDA black box warning, also documenting two Major Depressive Disorder patients whose refusal to undergo a pharmacological antidepressant treatment possibly led to an increased risk for suicidal behaviors. The concerns raised by the FDA black box warning need to be considered in real-world clinical practice, stating the associated clinical and public health implications.

## Introduction

Suicidal behavior (herein including completed suicide and non-fatal suicide attempts) accounts for remarkably high rates of mortality and morbidity worldwide. The World Health Organization (WHO) estimates that 800,000–1,000,000 individuals died by suicide in the year 2000, making it the 13^th^ leading cause of death that year (1).

Suicide may occur within the course of Severe Mental Illnesses (SMIs) such as Major Depressive Disorder (MDD), Bipolar Disorder (BD), or schizophrenia, as well as other conditions. Contributory factors for increased suicide risk include the presence of anxiety, personality and substance use disorders, severe neuropsychiatric disorders and head injury, inherent genetic factors, clinical psychological factors, aggression and impulsivity, hopelessness, history of childhood abuse, adverse life events and psychosocial stressors, just to name few (2, 3).

Suicide may occur as part of the natural course of SMIs, or it may occur due to improper treatment of severe depression (4, 5).

While sub-optimal dosage of the antidepressant drug (or exposure to the physical and psychosocial treatment) may intuitively represent inadequate treatment of depression (inflating the risk for suicidality), the operational definition of optimal treatment of depression remains elusive (4).

Several reports highlighted the risk associated with antidepressant drug monotherapy in BD (6–8). However, the corresponding evidence for MDD is not that compelling, especially beyond the acute treatment phase of unipolar depression (9).

Based on anecdotal reports suggesting an inflated rates of suicidal behavior among MDD patients exposed to the Selective Serotonin Reuptake Inhibitors (SSRIs) (10), beginning from October 2003, the U.S. Food and Drug Administration (FDA) issued a series of health advisories and warnings for children and adolescents prescribed with antidepressant drugs (11). Such a claim was supported by a subsequent, controversial meta-analytic report of industry-sponsored trials on pediatric patients (12). Notably, the analysis—carried out by the FDA on controlled clinical trials encompassing 2,200 children exposed to any of the nine FDA-approved antidepressants commonly prescribed at the time—could not highlight an overall difference in terms of suicide risk with antidepressants vs. placebo. Such analysis could not even highlight any difference across the appraised SSRIs or other types of antidepressants vs. placebo (13). Indeed, the FDA-conducted meta-analyses of 372 randomized clinical trials of antidepressants involving nearly 100,000 participants, which showed that rates of suicidal thinking or behavior was higher among patients assigned to antidepressants when compared with placebo, and in a subsequent age-stratified analysis it was shown that such increased risk was significant only among children and adolescents under the age of 18 years. There was no evidence of increased risk among adults older than 24 years, and, among adults 65 years of age or older, antidepressants had an apparent protective effect against the development of suicidal ideation and behavior. The meta-analysis performed by the FDA had some methodological issues. For example, the assessment of suicide in the trials included in the pooled analysis is questionable from a validity standpoint, since the appraised trials were not primarily designed to assess suicidality as the primary outcome prospectively.

Moreover, how the risk of an event is measured has significant implications. The fact that antidepressants increase the risk of suicide in people younger than 18 years old holds true. However, how this risk is presented affects the perception of the prescribing clinicians (and possibly the attitude of the patients handling the antidepressant booklet). When the risk of suicidality is defined in absolute terms, the impact is negligible; however, when it is defined in relative terms, using a measure such the odd ratio (OR), the risk seems to be magnified.

On October 2004, the FDA required a so-called black box warning for antidepressant drugs of any class. That warning became effective in January 2005. In 2006, the FDA warning extended to young adults aged up to 25 years, an announcement that followed a slew of media reports about the link between antidepressant drug use and suicide, possibly culminating in an exaggerated alarmist message (14).

The FDA black box warning on antidepressant-linked suicide in young people has been questioned (15), and recent evidence on the overall benefits from the use of antidepressants in the treatment of MDD seems not to be so pessimistic (16). Major concerns about the actual validity of the FDA black box warning by independent clinical researchers has accumulated over recent years, with some experts fearing that the “*black box warning would be the final step before prohibiting the use of medication*” (17).

*The over-concern raised by the FDA most likely proved to be effective in decreasing the prescription of effective medications*. The rates of antidepressant drug prescriptions declined nearly 50%, especially in children and adolescents (18). The rate of the diagnosis of MDD reduced almost 40% (19), possibly due also to the diagnostic shift in favor of BD within the past decade and the availability of the second-generation antipsychotics, SGAs, besides the regulatory issues. The anticipated increase in the use of face-to-face psychotherapy to treat depression and prevent suicide expected by the FDA did not occur (14, 19). On the contrary, the utilization rates of psychoactive drugs such as benzodiazepines, and antipsychotics increased over the time following the FDA black box warning, especially among young females (15). However, it is worth noticing that even if suicide or some other adverse outcome had worsened in 2005 onward, coincident with a decline in antidepressant use, it would not necessarily mean that the two phenomena were connected to each other (20). Despite the increasing body of evidence contrasting the strict regulatory rules enforced by the FDA, the agency seems reluctant to retract the black box warning (14), even if that claim may rely on miscalculations, or it may not fit the real-world practice, as documented in the case reports outlined in the following lines.

## Case Reports

### Patient n.1

Mrs. A. is a 24-year-old married Caucasian woman denying any personal or familial history for psychiatric disorders or other medical conditions.

On May 2014, Mrs. A. reported intense psychological and physical discomfort for the first time in her life, beginning soon after she lost her job, and she experienced significant financial problems. The patient sought professional support by a private practice psychiatrist, who prescribed the following: escitalopram up to 15 mg/day and alprazolam 0.75 mg/day. Despite exhaustive consulting and multiple efforts towards an optimal doctor-patient relationship, the patient refused to start her treatment, even at low doses and despite the offer for more frequent, yet still affordable, follow-up visits. Upon inquiring about her refusal, the patient expressed significant concerns related to the information she came across over the internet and the booklet enclosed in the escitalopram box. Regrettably, the patient’s beliefs were supported by her husband, who questioned the actual efficacy of the antidepressant drug (besides the safety concerns already raised by his wife). The patient strongly opposed the prescription made by the treating physician, so she ended up taking just alprazolam, a benzodiazepine, to self-medicate her pain. She did not inform her psychiatrist about her partial adherence to the prescribed drugs. The patient also missed most of the follow-up visits, so that the treating clinician had no chance to prevent or to foresee her first suicidal attempt, which occurred about three months after she initially sought psychiatric consultation.

The patient attempted suicide by jumping out of her home window. Such an attempt was averted by her husband, who rushed the patient to the E.R. unit of the Teramo public hospital (Italy) soon after that. The psychiatrist on duty at the hospital confirmed the diagnosis of MDD made by the private practice physician, confirming the escitalopram prescription (also increasing the dosage up to 20 mg/day), and alprazolam. The consultant psychiatrist reassured both the patient and the husband and pointed out the risks associated with untreated depression, including suicidal behavior, especially after previous attempts. The therapist made intense efforts to explain to the couple the relevance for balanced evaluations of the efficacy, safety and tolerability profile for each drug, and to trust the physician irrespective of the advertisement posted elsewhere. Since the patient refused admittance to the inpatient unit, she was discharged, closely monitored and evaluated every week (free consultations offered in the public healthcare system), with the specific goal of building up a better patient-doctor relationship to finally achieve recovery.

After roughly two months, psychoeducation and most likely regular intake of escitalopram, led to substantial remission of depression.

Moreover, the patient did not show any adverse effect or worsening of suicidal ideation due to antidepressant therapy in the long-term period (last evaluation carried on September 2016). The patient self-reported to regularly take escitalopram 10 mg/day upon achieved euthymia, joining a mutual aid self-aid group run by patients who recovered from depression, being also active in arranging meetings and encouraging people to seek help and taking the prescribed medications, possibly developing some sort of proactive reactive formation during the follow-up visits.

### Patient n.2

Mr. S. is a 23-year-old Caucasian man with a three-year history of panic disorder (PD). He was a semi-professional football player before experiencing his first panic attack on June 2013, while driving through into a tunnel. His familial and personal history was negative for any psychiatric disorder, including substance abuse or suicide. He sought several medical consultations (i.e., cardiologist and neurologist) before reaching a psychiatrist who prescribed paroxetine gradually up-titrated up to 20 mg/day and alprazolam 0.50 mg/day for his newly diagnosed PD. The patient took paroxetine only for a few days as he was skeptical about its efficacy and concerned about the black box warning on antidepressant-related suicide.

Moreover, Mr. S. reported that several internet websites and forums he consulted were highly informative about the hazards of exposure to antidepressants and the related induction of both tolerance to antidepressants and suicide. Similar beliefs were held by some of his close peers and friends.

Within the following six months, the patient suffered several further panic attacks, refusing to take medication regardless of the efforts made by his treating psychiatrist to reassure him. As a consequence, the number and the frequency of new panic attacks surged within the following months. Intense panic attack recurrence occurred roughly ten times a week (usually twice a day) when he finally started to take alprazolam (still misreporting to his doctor about paroxetine due to the black box warnings and the fear to commit any involuntary suicidal behavior).

The patient quickly developed tolerance to alprazolam, ending up in abuse of daily doses up to 5mg. When finally dismissed by his football team without appeal owing to his now-evident addictive behavior towards benzodiazepines, he developed clinical depression, while still suffering panic attacks. He was then evaluated by another psychiatrist who confirmed the paroxetine prescription, which the patient refused to take due to his strong concerns about increased risk for suicidality, regardless of the additional efforts made by the psychiatrist to reassure and properly inform him under careful monitoring over the time. The patient did not show up to follow-up visits. Mr. S. attempted to commit suicide hanging from his bathroom doorknob when rescued by his brother.

The patient was rushed to the E.R. service of a hospital in Central Italy, where he was evaluated by a consultant psychiatrist who confirmed the diagnosis of MDD with comorbid PD. The prescribed therapy included the following: paroxetine (up to 20 mg/day), progressive down-titration of alprazolam to 3 mg/day and sodium valproate (500 mg/day). The patient was critical about his attempt since his first hospital admission, accepting to take the prescribed medications after full disclosure of both the pros and cons of antidepressant therapy, at the presence of his relatives, who possibly acted as a deterrent for new infringement of therapeutic recommendations. Indeed, the patient started to take the therapy regularly and, after a couple of weeks, he experienced substantial remission of symptoms, achieving full symptom remission within two months. Alprazolam and valproate were gradually tapered before being stopped in full with no inconvenience for the patient. The last observation was carried on October 2016, and the patient was entirely asymptomatic while taking only paroxetine 20 mg/day without reporting any adverse effects, not even hypomania. At that time, the patient restarted playing in a football team, also serving as a volunteer in an association helping patients with psychiatric disorders.

*Both patients provided valid informed consent prior to our presenting this piece of work*.

## Discussion

The two case reports briefly presented herein add to the relatively scarce evidence available in the literature about the potential consequences of over-concern raised by the FDA black-box warning on antidepressant and suicide.

The two case reports presented highlight the following: i) Misleading interpretations about the black-box warning persist after roughly a decade since the FDA 2007 revision. Leisure activity (i.e., sport), stable familial relationships, and average education level are not sufficient deterrents. Paradoxically, higher education, and chances to access informed web resources may backfire. ii) Most psychiatrists still face strong stigma and opposition by some patients and their relatives, or peers. iii) Adherence and physician trust are undermined by the overwhelming information available through the media.

Almost every year or so, another pop therapist comes along to persuade depressed people that they should soldier on with their misery rather than try antidepressants and that most antidepressants are either quackery or just harmful prescriptions pushes by pharmaceutical companies. While well-grounded, evidence-based awareness about the potential risk of antidepressant-induced risk for suicide in a minority of depressed cases is a necessary reminder, and the FDA needs to continue to ensure information on the topic, a severe warning may sound like an alarm.

Both cases briefly presented herein raise some issues which should deserve a critical appraisal. Among others is the issue above of stigma, which is still a significant concern in modern clinical psychiatry and relates to deep cultural factors shared among different social groups and cultural backgrounds (21). Also, adherence is already weak in most severe cases of depression, as these are patients who rarely spontaneously seek for medical care, not even when experiencing sudden and very intense worsening of overall symptomatology (as depicted in case n.2). Similarly, polypharmacy and the lack of a multi-disciplinary approach, as well as a propensity for benzodiazepine abuse, are well-known phenomenon that essentially regard full-threshold bipolar disorder (BD) patients (22, 23). Yet this could also be extended to some DSM-IV-defined cases of MDD (24), whereas the therapeutic alliance would become crucial especially for long-lasting treatments (25).

Both patients exhibited affective temperament traits suggestive of cyclothymia, which in turn would point towards a bipolar diathesis (26). This is with reference to the plausible demonstrative acts carried out by patient n.1, or the intense stress experienced by patient n. 2 in response to interpersonal and social rejection. However, none of the two patients underwent affective temperament nor sub-threshold bipolarity assessment (27, 28).

The lack of use of either the Mood Diagnostic Questionnaire or the Hypomania Check-List-32 represents a significant issue in our reports, especially considering that local language adaptations have been made available at the time of the patients’ evaluation (29–31).

Specifically, patient n. 2 showed two signs suggestive of sub-threshold bipolarity of the otherwise diagnosed DSM-IV MDD: lifetime PD and rapid pop-up response on antidepressants (32, 33) as seldom occurs in DSM-IV-defined full-threshold BD with current MDE (34). Yet, there is no FDA recommendation about the need to systematically assess sub-threshold bipolarity to date.

Although suggestive and relevant from a clinical (6) and a methodological standpoint (35), the actual potential impact of sub-threshold bipolarity in connection with suicidal ideation due to antidepressant exposure in some MDD cases is beyond the possibility of the present concise report. Indeed, neither of the two patients exhibited overt manic switch or any over mixed features within the follow-up period. Nonetheless, both cases paradoxically document how neglected sub-threshold bipolarity assessment is in the clinical practice, including primary care (36).

It is also worth noticing that pivotal pharmaco-epidemiologic studies found a relationship between a higher number of sales or prescriptions of antidepressants and a lower suicide rate (37–39).

Warnings that antidepressants may increase suicides appear to have backfired as suggested by Rihmer (40, 41) and by Isacsson and Allner (42), who analyzed a database of 845 suicides in the 10- to 19-year age group in Sweden within the years of 1992–2003 (baseline) and 2004–2010 (after the warning), concluding that “…the warning, contrary to its intention, may have increased young suicides by leaving a number of suicidal young persons without treatment with antidepressants.” Moreover, the presence of the black box concerning suicidality had a great resonance on several internet websites and forums, with dangerous amplification of the perceived risk, especially for those who have no real scientific basis, as was the case with both patient n. 1 and n. 2. In particular, patient n. 2 was overconcerned about the risk of developing tolerance to antidepressants, a scenario which albeit infrequent, may inflate the rates of recurrence of depression and suicide (33, 43), thus prompting further assessment by the FDA (44, 45).

Among other implications, the black box warning issued by the FDA could not determine any conclusive causal relationship. Depression represents a trans-diagnostic condition, and this is also why the antidepressant drug nomenclature seems outdated and misleading (because of its disease-targeted approach) in contrast to the novel neuroscience-based nomenclature, which seems more sound (46).

Milane et al. (47) found an inverse correlation between suicide rates and fluoxetine prescriptions (47). Re-analysis of published data by Gibbons et al. (48) found that fluoxetine and venlafaxine decreased suicidal thoughts and behavior for adults. Once again, this may suggest a possible differential developmental neurobiological profile among the cases that will be harmed by antidepressants, especially during adolescence or young adulthood vs. other cases which may benefit. Coupland et al. (49) demonstrated higher rates of suicide/suicide attempt/self-harm just after starting/stopping antidepressant treatment, concluding that, overall, treatment with antidepressant(s) may improve outcomes (49) given that depression itself is inherently associated with an increased risk of suicide, suicidal behavior, anxiety and pharmaco-phobia overall (50).

Above all, based on the present case reports, we suggest that the black box should finally be lifted, at least for adult patients, at least until further light can be shed over such an inconclusive and vividly debated matter.

We believe that the Hippocrates’s principle “*primum non ncēre*” (“*first not harm*”) should be accepted without any prejudice. In other words, while the potential risk of induced suicide caused by antidepressants in children or adolescents or adults serves as a prompt for careful vigilance, the risk for over-concern among clinicians and patients, and the subsequent actual risk for suicidality due to the progression of the natural course of MDD, should likewise be appropriately counterbalanced.

The risk of suicide associated with untreated depression or anxiety disorder is high and represents a significant public health concern (51). As pointed out by Friedman (52), the FDA was aware of the need to balance the quantitatively small risk associated with antidepressant treatment against related proven benefits, yet it expanded the black-box warning issued in 2007 stating that depression itself is associated with an increased risk of suicide (52). Roughly ten years have passed since the extended warning by the FDA and the subsequent alarm from some prescribing clinicians over-concerned about regulatory, license or insurance issues, as well as by patients or relatives exposed to stigmatization or incorrect information (53).

Also, the FDA may have accumulated additional data over the past decade, yet not allowed it to be released to the general public, potentially affecting the judgment of risk.

Interestingly, in the year 2008, the FDA made an alert about antiepileptic drugs (AEDs), stating that they may increase the risk of suicide. In that occasion, the scientific advisory committee voted against placing a black box warning on AEDs and suicide, although there was a significant positive association between AEDs and suicidality. The FDA attitude towards the AEDs may be perceived as a change in the position of the FDA over time, soliciting revision and updating of the black box label warning for antidepressant drugs.

The FDA expanded the black box warning already in the year 2007, stating that depression carries an increased risk of suicide itself. Further revision is warranted (54), placing further emphasis on the physician-patient interaction and the involvement of the caregivers, especially during the initial phase of the antidepressant treatment to reduce the risk for emergent suicidal ideation and self-harm.

## Author Contributions

DD recorded the clinical information relevant to the present report. DD and MF drafter the manuscript and its subsequent revisions, while all fellow co-authors assisted either in the critical interpretation of the findings and/or in the literature review.

## Conflict of Interest Statement

The authors declare that the research was conducted in the absence of any commercial or financial relationships that could be construed as a potential conflict of interest.
